# Eltrombopag for the Treatment of Immune Thrombocytopenia: The Aegean Region of Turkey Experience

**DOI:** 10.4274/tjh.2014.0152

**Published:** 2015-12-03

**Authors:** Füsun Özdemirkıran, Bahriye Payzın, H. Demet Kiper, Sibel Kabukçu, Gülsüm Akgün Çağlıyan, Selda Kahraman, Ömür Gökmen Sevindik, Cengiz Ceylan, Gürhan Kadıköylü, Fahri Şahin, Ali Keskin, Öykü Arslan, Mehmet Ali Özcan, Gülnur Görgün, Zahit Bolaman, Filiz Büyükkeçeçi, Oktay Bilgir, İnci Alacacıoğlu, Filiz Vural, Murat Tombuloğlu, Zafer Gökgöz, Güray Saydam

**Affiliations:** 1 Katip Çelebi University Faculty of Medicine, Atatürk Research and Education Hospital, Clinic of Hematology, İzmir, Turkey; 2 Ege University Faculty of Medicine, Department of Hematology, İzmir, Turkey; 3 Pamukkale University Faculty of Medicine, Department of Hematology, Denizli, Turkey; 4 Bozyaka Research and Education Hospital, Clinic of Hematology, İzmir, Turkey; 5 Aydın State Hospital, Clinic of Hematology, Aydın, Turkey; 6 Dokuz Eylül University Faculty of Medicine, Department of Hematology, İzmir, Turkey; 7 Tepecik Research and Education Hospital, Clinic of Hematology, İzmir, Turkey; 8 Adnan Menderes University Faculty of Medicine, Department of Hematology, Aydın, Turkey

**Keywords:** Immune thrombocytopenia, Thrombopoietin receptor agonist, Bleeding, Eltrombopag

## Abstract

**Objective:**

Immune thrombocytopenia (ITP) is an immune-mediated disease characterized by transient or persistent decrease of the platelet count to less than 100x109/L. Although it is included in a benign disease group, bleeding complications may be mortal. With a better understanding of the pathophysiology of the disease, thrombopoietin receptor agonists, which came into use in recent years, seem to be an effective option in the treatment of resistant cases. This study aimed to retrospectively assess the efficacy, long-term safety, and tolerability of eltrombopag in Turkish patients with chronic ITP in the Aegean region of Turkey.

**Materials and Methods:**

Retrospective data of 40 patients with refractory ITP who were treated with eltrombopag in the Aegean region were examined and evaluated.

**Results:**

The total rate of response was 87%, and the median duration of response defined as the number of the platelets being over 50x109/L was 19.5 (interquartile range: 5-60) days. In one patient, venous sinus thrombosis was observed with no other additional risk factors due to or related to thrombosis. Another patient with complete response and irregular follow-up for 12 months was lost due to sudden death as the result of probable acute myocardial infarction.

**Conclusion:**

Although the responses to eltrombopag were satisfactory, patients need to be monitored closely for overshooting platelet counts as well as thromboembolic events.

## Abstract (tr)

**Amaç:**

İmmün trombositopeni (İTP), trombositlerin immün aracılı yıkım ile kalıcı veya geçici olarak 100x109/L altında olduğu bir hastalıktır. Selim hematolojik hastalıklar içinde yer almasına rağmen kanama komplikasyonları ölümcül olabilir. Hastalığın patofizyolojisi daha iyi anlaşılması ile son yıllarda kullanıma giren trombopoetin reseptör agonistleri, dirençli hastaların tedavisinde etkili bir seçenek olarak görünmektedir.

**Gereç ve Yöntemler:**

Bu çalışmada Ege Bölgesi’nde refrakter İTP tanısı ile eltrombopag ile tedavi edilen 8 farklı merkezden 40 hastanın retrospektif verileri incelenmiş ve değerlendirilmiştir.

**Bulgular:**

Çalışmada toplam yanıt oranı %87 idi ve trombositlerin 50x109/L’nin üzerine çıktığı medyan süre 19,5 (5-60) gün saptandı. Tromboz için başka hiçbir ek risk faktörü bulunmayan bir hastada venöz sinüs trombozu gözlendi. On iki aydır tam yanıtlı izlenen ve düzensiz takibe gelen bir diğer hasta olası akut miyokard infarktüsü sonucu gelişen ani ölüm nedeni ile kaybedildi.

**Sonuç:**

Her ne kadar eltrombopag yanıtları tatmin edici olsa da, hızlı ilerleyen trombositemiye bağlı gelişecek tromboembolik olaylar açısından, yakın takip ve monitorizasyon gereklidir.

## INTRODUCTION

Immune thrombocytopenia (ITP) is an acquired autoimmune disease in which antiplatelet antibodies accelerate the destruction of platelets in the reticuloendothelial system and is characterized by impaired platelet production, resulting in low platelet counts [1]. Among adults, about 50 new cases of ITP per million are diagnosed per year [2]. In adults, the course of the disease is commonly chronic. The primary goal of treatment is to prevent bleeding by increasing the platelet count to a stable level while managing the few treatment-related toxic effects. Current guidelines suggest that treatment should only be considered in symptomatic patients with platelet counts of less than 30x109/L. Treatment is rarely indicated for patients with platelets of >50x109/L in the absence of bleeding or predisposing comorbid conditions [1,3]. The first-line treatment for ITP is glucocorticosteroids. For patients who are actively bleeding or who have a contraindication to glucocorticosteroids, intravenous immunoglobulin or anti-D globulin can be used [4]. These drugs increase platelet counts primarily by reducing the extent of platelet destruction by several different mechanisms. In the case of glucocorticosteroid treatment failure, splenectomy is the main second-line therapy and induces a 70%-80% response rate [5]. Until recently, in patients who were refractory to or relapsing after splenectomy or when splenectomy was contraindicated, a variety of immunosuppressive or cytotoxic drugs (such as vincristine, cyclophosphamide, azathioprine, cyclosporine A, and rituximab) were common as the third-line therapy. However, almost 30% of adults with ITP fail to respond to these therapies and eventually develop a chronic refractory disease [2,6,7]. All of these treatments mainly reduce destruction of antibody-coated platelets; however, treatment is not always effective and can be restricted by adverse effects. ITP is often considered as benign disorder, but health-related quality of life is poor. Most of the treatment strategies, such as glucocorticosteroids and immunosuppressive drugs, adversely affect quality of life. In recent years, a better understanding of the pathophysiology of ITP has demonstrated the impaired thrombopoiesis and has led to the development of new therapeutic approaches. A new approach to the treatment of ITP is based on platelet production rather than destruction of platelets. Eltrombopag is an oral, nonpeptide, thrombopoietin receptor (TPO-R) agonist, approved in several countries for the treatment of chronic ITP. Eltrombopag increases platelet production by interacting with the transmembrane domain of the TPO-R and inducing proliferation and differentiation of bone marrow progenitor cells in the megakaryocyte lineage [8,9]. It can be prescribed in Turkey since November 2011. In this study we aimed to retrospectively assess the efficacy, long-term safety, and tolerability of eltrombopag in Turkish patients with chronic ITP in the Aegean region of Turkey.

## MATERIALS AND METHODS

This study was designed as a retrospective study. A total of 40 patients who received eltrombopag treatment for refractory chronic ITP at 8 different centers in the Aegean region of Turkey were included.

ITP diagnosis was verified according to the International Consensus Report on the Investigation and Management of Primary ITP [1]. Primary ITP requires only the finding of isolated thrombocytopenia (100x109/L) with no obvious associated medical condition [1]. Patients were aged 18 years and older and had primary ITP of more than 6 months’ duration, had baseline platelet counts of lower than 30,000/μL, and had relapsed after one or more previous treatments for their disorder. The form prepared for the study was sent to all centers. Date of the first diagnosis of the patients, demographic data, time to splenectomy, previous treatments and response to treatments, side effects, posttreatment follow-up period, and other such records were retrospectively evaluated. The most recent patient data were recorded in December 2013. Bleeding was assessed with the World Health Organization bleeding scale (grade 0: no bleeding, grade 1: petechiae, grade 2: mild blood loss, grade 3: gross blood loss, grade 4: debilitating blood loss) [10]. Response rates were defined as follows: complete response when the platelet count was 100x109/L, partial response when the platelet count ranged between 30 and 100x109/L with at least a 2-fold increase in the initial platelet count, and no response when the platelet count was 30x109/L [3].

### Statistical Analysis

Statistical analysis was performed using SPSS 18.0 (SPSS Inc., Chicago, IL, USA). The Kolmogorov-Smirnov test was used to evaluate the distribution of data. Data with normal distribution were reported as mean ± standard deviation (SD), while data with nonnormal distribution and nonparametric data were reported as medians (interquartile ranges, 25%-75%). To evaluate effect of baseline platelet counts on treatment by eltrombopag, the Mann-Whitney U test was used. For comparison of categorical variables, Pearson’s chi-square test was used, or in the case of small frequencies, Fisher’s exact test was used. Statistical significance was defined as p<0.05.

## RESULTS

**Figure 1:**
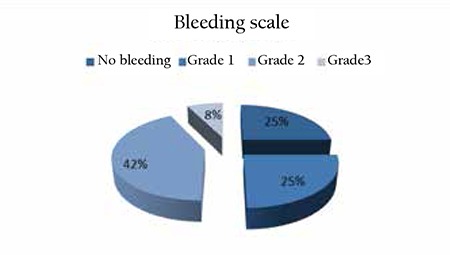
Bleeding data of the patients prior to eltrombopag treatment.

**Figure 2:**
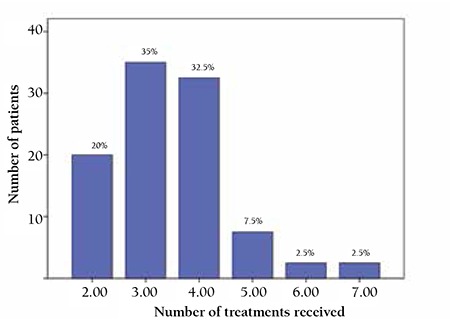
Number of treatments prior to eltrombopag.

**Figure 3:**
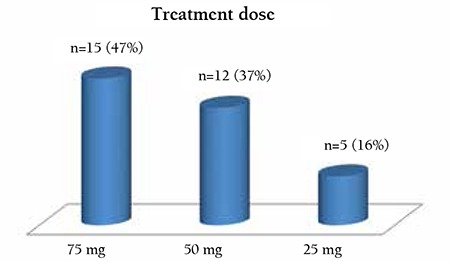
Eltrombopag requirement of patients.

In total, 40 patients, 30 of them women, from 8 centers in the Aegean region of Turkey were included in the study. All of them had received glucocorticosteroids at various doses as first-line treatment, and splenectomy was implemented in 28 cases as second-line treatment due to resistance against steroid treatment. Splenectomy was not implemented in 12 cases because of incompatibility for surgery due to comorbid disease or the patient’s disapproval. Prior to eltrombopag treatment, bleeding scale scores were evaluated for each patient and previous treatment numbers were revised and recorded (Figure 1). Median previous treatment number was found to be 3 (interquartile range: 3-4) (Figure 2). Eltrombopag was initiated at 50 mg/day for all patients and the dose was regulated in accordance with their response to treatment. The distribution of the last drug dose is given in Figure 3. Treatment was stopped in 5 cases since no response was obtained. Two patients were lost due to intracranial hemorrhage in the first month of the treatment. Patient characteristics are reported in Table 1.

Mean platelet count before treatment was 11.5x109/L±8.3x109/L. The total rate of response was 87% and in the cases with response the median period in which the number of platelets reached over 50x109/L was determined as 19.5 (interquartile range: 5-60) days (Table 2).

As for response to treatment, whether or not there was any sex-related difference was studied by chi-square test; no significant difference was determined for response to treatment (p=0.629). Whether the baseline platelets had any significance in patients’ response to treatment was analyzed by Mann-Whitney U test and it was seen that baseline platelet count was not important in response to treatment (p=0.531). From the viewpoint of response to eltrombopag treatment, the patients having had splenectomy and those who had not were compared by chi-square test. No difference was determined between the patients with and without splenectomy in their response to eltrombopag treatment (p=0.370).

In the 21 cases in which bone marrow biopsy was done prior to treatment, bone marrow reticulin was evaluated in 2 cases as 2, in 3 cases as 1, and in 16 cases as 0. During treatment, none of the patients showed any clinical or laboratory findings suggesting increased bone marrow reticulin and bone marrow biopsy was not repeated. Adverse effects due to treatment are summarized in Table 3. Of the cases with response to treatment, drug-related nausea developed in 2 cases and headache in 4 cases. However, drug use was continued and these adverse effects vanished in a few weeks after the beginning of the treatment. Platelet count was below 50x109/L on the 7th day of treatment in a case in which erythromelalgia developed, whereas on the 13th day it reached 580x109/L. However, it receded back to the baseline value about 2 weeks after termination of drug use. The patient was suggested to start the drug again with a lower dose but refused the treatment. One male patient at the age of 35, having venous sinus thrombosis and showing no other additional risk factors from the point of view of thrombosis, was receiving 50 mg eltrombopag and had a platelet value on the 15th day of 680x109/L. Treatment was terminated. Platelet counts receded back to below 10x109/L in 15 days; due to recurrent epistaxis and intraoral bleedings, treatment was resumed with dose regulation and no thrombotic attack was observed.

In another patient, treatment was stopped due to an increase in transaminases. Transaminase levels were all in normal ranges prior to eltrombopag therapy. Alanine transaminase (ALT) and aspartate transaminase (AST) levels of this patient had gradually increased while she was on eltrombopag. After the ALT level had reached up to 3 times the upper normal level, eltrombopag was stopped with a presumptive diagnosis of toxic hepatitis possibly related to eltrombopag. In order to clarify the etiology of elevated transaminases and to be certain about whether this co-incidence was a side effect of eltrombopag or was another concomitant disease, hepatitis serology and autoimmune tests were applied. Serology results were all negative considering hepatitis A, hepatitis B, and hepatitis C. Antimitochondrial antibody was positive with a titer of 1/1000. Liver biopsy was applied for further clarification of ongoing transaminitis and it revealed autoimmune hepatitis. The elevated transaminases were therefore not considered as a side effect of the drug, rather being considered as an independent concomitant autoimmune disorder. After proper treatment of autoimmune hepatitis with steroids and azathioprine, transaminase levels decreased to normal ranges. At the same time, platelets counts were at a steady level between 50,000 and 70,000/µL with the aforementioned immunosuppressive therapy and eltrombopag was not reinitiated.

One of the patients with complete response who was followed irregularly for 12 months was lost due to sudden death as a result of probable acute myocardial infarction. In the laboratory tests performed on the same day, the patient’s platelets were measured as 120x109/L. Two different patients who both had complete response at the beginning of treatment but whose platelet counts decreased below 10x109/L in the 8th month and 1st year of treatment were found to be taking iron supplements and calcium supplements, respectively. These patients were warned about drug and diet interactions, and their platelet counts increased again to above 100x109/L in further follow-up.

Target platelet counts after therapy should be between 50 and 100x109/L, not normalization. In 2 patients, despite platelet counts of 30 to 35x109/L, treatment continued with partial response since bleeding symptoms were controlled.

One patient for whom 4 different treatment options had been previously applied with no response, and who was progressing with intraoral bleedings recurring frequently, received a splenectomy in the 2nd month of eltrombopag treatment while platelet counts were over 100x109/L. The patient then started follow-up with complete response without treatment. Treatment doses in responding patients are given in Figure 3. In the patients with response to treatment, average follow-up period was evaluated as 13.78±7.51 months.

## DISCUSSION

In this retrospective study, we have evaluated the long-term safety, efficacy, and tolerability of eltrombopag use on Turkish patients with chronic ITP. In this study in which the data of 40 patients were evaluated retrospectively, the total rate of response is 87%, where various different treatment options such as steroids, anti-D globulin, splenectomy, intravenous immunoglobulin, azathioprine, cyclophosphamide, danazol, vincristine, and rituximab were applied with no response prior to eltrombopag treatment. This rate was similar to the rate of 80% obtained in the study of Katsutani et al., where 3 years of eltrombopag data from 19 patients were evaluated, and to the rate of 69.6% obtained in the study of Tomiyama et al. including 23 patients with a placebo control [11,12]. In the present study, there was no difference between the response rates of patients with and without splenectomy, in accordance with the literature [13,14]. Although the responses were satisfactory, patients need to be monitored closely regarding rapidly progressing thrombocythemia as well as thromboembolic events.

Treatment was generally well tolerated and continued, except for a patient who developed erythromelalgia in the 1st month of therapy and another patient who developed autoimmune hepatitis in the 6th month. While eltrombopag is known to have the ability of increasing transaminases [15], treatment was terminated in the patient who developed autoimmune hepatitis. However, during follow-up, no decrease in transaminases occurred despite discontinuing the drug. This situation was thus regarded as a concomitant disease.

The common side effects of eltrombopag treatment, headache and nausea, did not cause any termination in the treatment and disappeared spontaneously over time. In the literature, the incidence of thromboembolic events was reported as 2%-4% during treatment with TPO-R agonists; however, the rate of only 1 patient out of 40 having sinus vein thrombosis was consistent with the literature [16]. We could not obtain detailed information about the patient who was lost to acute myocardial infarction in the 12th month of treatment while being monitored with full response.

TPO-R agonists may increase the risk of developing or progressing reticulin fiber deposition in the bone marrow [17]. For patients on eltrombopag, peripheral blood smears should be examined for morphological abnormalities such as teardrop cells, nucleated red blood cells, leukoerythroblastic pictures, dysplastic changes, or cytopenia [18]. If such abnormalities develop or deteriorate, a bone marrow biopsy should be performed. A loss of response or failure to maintain a platelet response with eltrombopag treatment within the recommended dosing range should also prompt a search for causative factors such as myelofibrosis [18]. In our study, no patients displayed suggestive clinical or laboratory findings of significant increases in bone marrow reticulin during treatment and bone marrow biopsy was not repeated. The average follow-up period in the patients who responded to treatment of 13.78±7.51 months was satisfactory; on the other hand, close monitoring is recommended for thrombocythemia and thromboembolic events. In particular, patients who begin treatment should be informed in this regard in detail, and this therapy is not recommended for cases that cannot be followed closely. A decrease in eltrombopag dosage is recommended when platelet counts exceed 200x109/L and should be completely stopped if platelet count is over 400x109/L. After discontinuation due to thrombocythemia or any other adverse effects, patients should be monitored to detect any transient decrease in platelet counts and to decide about further treatment indication and dose. In the case of response loss during follow-up in patients with an initial response, diet-drug interactions must be questioned in detail.

## Figures and Tables

**Table 1:**
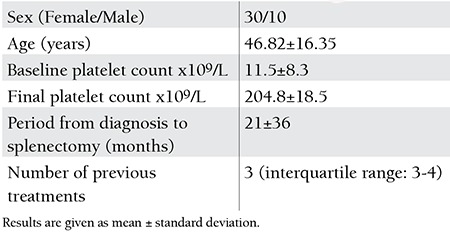
Baseline characteristics of the patients.

**Table 2:**
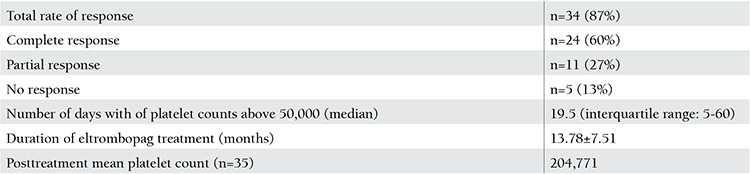
Outcomes of the treatment.

**Table 3:**
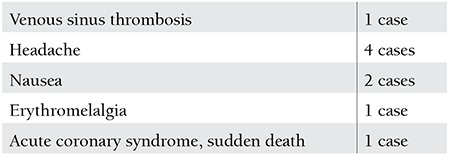
Adverse effects and toxicity of treatment.
